# Reply to “Height-related changes in forest composition explain increasing tree mortality with height during an extreme drought”

**DOI:** 10.1038/s41467-020-17214-4

**Published:** 2020-07-07

**Authors:** Atticus E. L. Stovall, Herman H. Shugart, Xi Yang

**Affiliations:** 1 NASA Goddard Space Flight Center, 8800 Greenbelt Rd., Greenbelt, MD USA; 2 University of Virginia, 291 McCormick Rd., Charlottesville, VA USA

**Keywords:** Climate-change ecology, Forest ecology

**Replying to** N. L. Stephenson & A. J. Das Nature Communications 10.1038/s41467-020-17213-5 (2020)

Recently, we published a study^1^ tracking tree mortality through an extreme drought for ~1.8 million individual trees over 8 years, revealing a continuous upward trend in mortality risk with respect to tree height. In the accompanying paper, Stephenson and Das dispute our findings, highlighting two scenarios in which broad changes in forest composition control mortality trends. We re-analyze our full tree-level dataset^2^, controlling for forest type by testing for an increasing height-mortality trend in ten unique topographic positions and ten unique forest types (Fig. 1). In all topographic positions and all forest types covered in the original 40,000-ha study area, we still find a consistent upward trend in mortality rate with increasing tree height. We also find that plot-based sampling schemes may not confidently detect the full height-mortality trend due to undersampling of tall trees in forests. Our remote sensing-based approach helps solve this logistical challenge. With these lines of evidence, alongside our original findings^1^, we argue in favor of a broad height-mortality trend that is interactive and modulated by species-specific factors. Drought-induced tree mortality is controlled by a complex series of interacting stressors—not by a single binary factor.

**Fig. 1:**
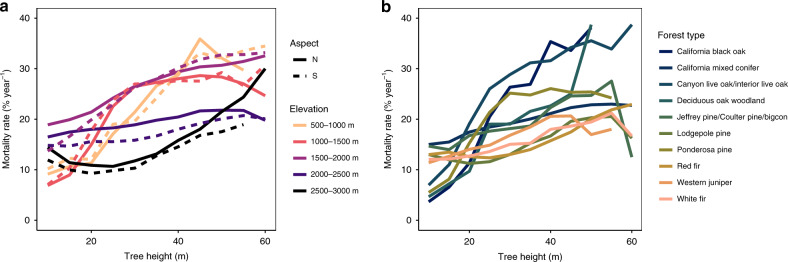
Consistent trends in mortality rate and tree height. Mortality rate continuously increases with height across all **a** topographic positions (2 aspects × 5 elevation bins) and **b** forest types in the study area. If broad shifts in canopy species are the primary cause of different mortality rates, we would expect to see disparate trends in mortality rate with increasing tree height in different forest types.

In each unique topographic position and forest type, a single consistent trend emerges: mortality rate increases with tree height (Fig. 1, See “Methods”). South-facing slopes have lower mortality rates than North-facing slopes across most elevation bands, potentially due to more drought-tolerant species groups occurring on drier, hotter mountain slopes. As discussed in our original study^1^, the broad differences in the mortality–height trend in these unique topographic positions interact with environmental gradients of vapor pressure deficit, temperature, and precipitation. While forest type controls the specific shape and sensitivity of the mortality–height relationship (mean = 4.9% yr^−1^; σ = 2.3% yr^−1^), every single forest type evaluated in this study area has a clear increase in mortality rate with increasing tree height (mean = 3.7% yr^−1^ m^−1^). If our broad result—height predicts mortality risk during drought—arose from shifting taxonomic groups alone, we would expect to find inconsistent relationships in the directionality of the height–mortality trend in differing forest types. In essence, this new result highlights the striking consistency in this height–mortality relationship across all ten different forest types in the 40,000-ha study area.

Discrepancies between the two studies in extent and sample size may explain their different findings. In comparison to our study^1^, their work^3^ has 0.02% of the total study area, ~14% of the elevation extent and ~0.5% of the total (*n* = 5855), and ~0.1% of the total trees in the largest height-class (>30 m; Stephenson and Das: 481 trees; this study: 600,509 trees). To infer mortality trends using relatively few large tree measurements, Stephenson and Das aggregates into broad taxonomic groups and height classes—potentially obscuring continuous landscape-scale relationships. In addition, we directly measure tree height—the key physical dimension capturing hydraulic limitation—with airborne laser scanning, while Stephenson and Das indirectly estimate tree height from diameter using allometric equations; Uncertainty in height predictions increases dramatically with diameter class. Given the massive effort in collecting field data, significant areal and sample size discrepancies are reasonable, but, with field data alone, undersampling the largest height class is extremely likely; Stephenson and Das’ plot-based approach underestimates the proportion of large trees by half (Stephenson and Das: 8%; this study: 16%). We hypothesize inconsistent trends in past studies could be, in part, due to insufficient observations from these tall (>30 m) and rare trees.

Ground-based estimates of the height–mortality trend are highly uncertain due to plot placement, with errors typically increasing with tree height (Fig. 2). Using our full tree-level dataset^2^, we test the uncertainty in mortality–height estimates using an identical plot sampling approach as Stephenson and Das in 1000 different plot configurations (See “Methods”). On average, the plot simulations closely approximate the number of samples per height class in the ground-based data^3^ (Supplementary Fig. 2) and, though lower sample size or obscured understory trees can be an issue with remote sensing, small-tree mortality rates align (~10–20% yr^−1^) between studies. Across 1000 simulations, we found substantial variation (σ ≈ ±7% yr^−1^; CV = 52%; Supplementary Fig. 3) in the height–mortality trend due to plot placement and a tendency to underestimate—as opposed to overestimate—mortality rate (5th percentile of errors = −388%; 95th percentile of errors = 47%).

**Fig. 2:**
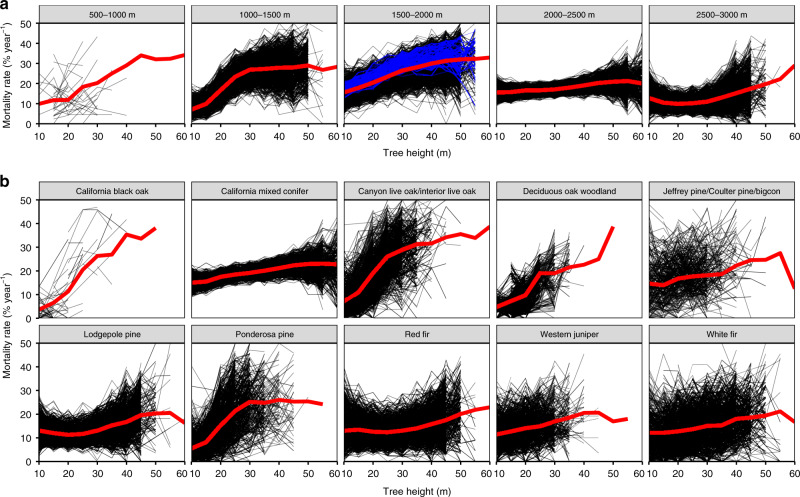
Simulated field plot sampling shows wide variability in mortality rate estimates. A single plot configuration (individual black lines) is statistically unlikely to capture the broad mortality-height trend (red lines) in **a** unique topographic positions or **b** forest types. We test 1000 different configurations of 89 0.1-ha plots in the 40,000-ha study area and (blue) a contiguous elevation range identical to Stephenson and Das, finding inconsistency in mortality–height trends is primarily due to random variation in plot placement.

Decreasing stability in the mortality-height trend with tree height is likely related to undersampling of the rarest and tallest trees in the forest. How rare are these trees? Our entire dataset of 1.8 million trees covering 40,000-ha of forest, had a mere 17 trees over 70 m tall and, on average, simulated plots sampled only 4 out of 3642 total trees identified in the 60 m class. Finding these rare trees is simply infeasible using traditional means—capturing a minimum of 10 trees taller than 60 m would require establishing ~1100 0.1-ha plots. Stephenson and Das correctly assert that “ground-based observations [should] support interpretation of remotely sensed data”, but, without remotely sensed data, ground-based observations are unlikely to fully capture landscape-level trends in rare trees. Given this high variability in mortality estimates relative to the landscape average, we conclude that any single plot configuration is unlikely to capture the broad mortality–height trend at the species level, but remotely sensed estimates of forest structure provide the necessary information to establish more effective plot networks.

Our broad conclusion that tree height increases mortality risk during drought^1^ and the scenarios presented in the accompanying paper are not mutually exclusive—they are interactive. While we agree that Stephenson and Das’ conclusions are logical and supported by their data, a vast body of literature supports the existence of hydraulic risk and the interactive nature of tree stressors^4–11^. For instance, while large trees are more hydraulically vulnerable^6^, they are also preferentially attacked by beetles, due to larger cambium tissue and thicker bark for overwintering^12^. Yet, beetles also preferentially attack hydraulically stressed trees^10,12^, which are more likely to be the tallest trees in the forest^6^. It is widely recognized that if the California drought had not been so extreme, mortality associated with beetles would not have been as severe^12^. We cannot accept the simple presence of beetles to be confirmation of mortality due to beetle attack, without understanding the internal hydraulic status of the tree. Neither study^1,3^ explicitly distinguished tree mortality by hydraulic failure, but given the complex of factors leading to mortality, attributing a single “cause of death” is far too one-dimensional.

The analysis shown here supports past work showing tree mortality is influenced by a complex set of well-established stressors and risk factors—including, but not limited to, tree height^1,4–6,10^, taxonomy^3,10,12^, geomorphology^1^, topography^1^, climate^1^, and pathogens^12^. Across ten different forest types, mortality risk consistently increases with tree height, suggesting stress due to hydraulic limitation may be a first-order control on mortality risk. Regardless of the critical role of hydraulic stress in tree death during drought, we do not attribute mortality to a single factor, but, instead, support a holistic view of mortality risk—considering a suite of interactive risk factors, ultimately leading to a tree’s death. Moving forward, massive-scale individual-based remote sensing will be critical in bridging the gap between the individual tree, inventory plot, and whole forest, revealing broader-scale trends in drought-induced mortality.

## Methods

### Classifying the tree-level dataset

The Sierra Nevada mountains have unique species assemblages following strong topographic gradients, with the most dominant shifts controlled by topography^12,13^. In the low elevations the canopy is dominated by pines, shifting to fir species in high elevations. South-facing slopes tend to be dominated by drought-tolerant species, while North-facing slopes hold species more suited for cooler, wet conditions. We control for species groups by testing the height–mortality trend on our full tree-level dataset (*n* = 1,808,334 total; *n* = 656,145 trees >30 m tall), subset by [1] broad topographic drivers of species, and [2] a more complex, modeled forest type map^13^. Using the topographic data calculated in our original analysis^1^, we categorize trees into ten topographic positions: the nearest 500 m elevation bands (500–2500 m) and the North or South-facing slope (270–90° and 90-270°, respectively).

Next, we associate the USFS Forest Inventory and Analysis (FIA) forest type map^13^ with each tree in our dataset^2^. The forest type map is a predictive map of forest type, based on every FIA plot available and a suite of environmental variables. Though the local accuracy of the forest type map is not exceptional (training = 89.5%; testing = 51.0%), especially along transitions, this approach should effectively capture broad swaths of similar species for the purposes of our analysis. In total, our study area has a total of 14 forest types, but we exclude 4, due to limited total area or a lack of data in multiple height classes, limiting our inference of the full mortality–height trend. While neither of these methods distinguishes species at the scale of tree crowns, they should be telling of height–mortality trends within this ecosystem’s common species groups.

### Simulating stratified plot-level data from the full dataset

We simulate 1000 different plot placement strategies using our full tree-level dataset^2^ and an identical plot sampling approach as Stephenson and Das. We stratified 89 circular 0.1-ha plots with a probability-based design using the categories defined in our prior analysis: [1] topographic position and [2] forest type. At each simulation step, 89 stratified plot locations are randomly selected using the *grts* function in the R package *spsurvey*. Total sample locations are determined by the proportion of area attributed to each topographic or forest type class. A simulated plot is created as a 0.1-ha subset of tree crowns identified in our original study^1^.

To ensure our plot-simulations were comparable to Stephenson and Das’ field data over the 5–60 m tree height range, we directly compare the average number of tree samples (dead and total) across all simulations with the field plot distributions with respect to height class (Supplementary Fig. 2). We also test the same plot simulation approach in a contiguous subset of our broad study area between 1524 and 1829 m in elevation, where mean slope was ~31° (compared to ~24° in Stephenson and Das’ study) with a standard deviation of ~13°. Of our subset area, 10% had steeper slope and, on average, contained more rock outcrops than Stephenson and Das’ field data. We quantified the distribution of errors in the plot-based simulations compared to the full landscape-scale mortality trend (Supplementary Fig. 3). In each population subset tested, errors were considered as a relative percent of the landscape-scale mortality rate for individual height-class and population subset.

### Reporting summary

Further information on research design is available in the Nature Research Reporting Summary linked to this article.

## Supplementary information

Supplementary Information

Reporting Summary

## Data Availability

The full tree-level dataset is available on figshare^2^ at: https://figshare.com/articles/CA_lidar_tree_mortality/7609193. The USFS Forest Inventory and Analysis (FIA) forest type map is available at: https://data.fs.usda.gov/geodata/rastergateway/forest_type/.

## Code availability

All code for the analysis in this work is available from the corresponding author upon request and will be uploaded to GitHub at: https://github.com/aestovall/tree_mortality.
