# Supplementary material for: Reply to “Height-related changes in forest composition explain increasing tree mortality with height during an extreme drought”
Source: Nat Commun. 2020 Jul 7;11:3401. doi: 10.1038/s41467-020-17214-4 (PMC7340790; doi:10.1038/s41467-020-17214-4)
Supplement: Supplementary file 1 — Supplementary Information [file 41467_2020_17214_MOESM1_ESM.pdf]

Supplementary Information

Title:
Reply to "Height-related changes in forest composition, not tree vulnerability, explain increasing mortality with height during an extreme drought"

Authors:
Atticus Stovall[1,2*], Herman Shugart[2] & Xi Yang[2]

Author Affiliations:
[1]NASA Goddard Space Flight Center, 8800 Greenbelt Rd., Greenbelt, MD, United States
[2]Department of Environmental Sciences, University of Virginia, 291 McCormick Rd., Charlottesville, VA, United States

Corresponding Author:
Atticus Stovall, Ph.D.
8800 Greenbelt Rd., Building 33
Greenbelt, MD, 20771
Phone: 301.614.6677
Email: atticus.e.stovall@nasa.gov

*Corresponding Author

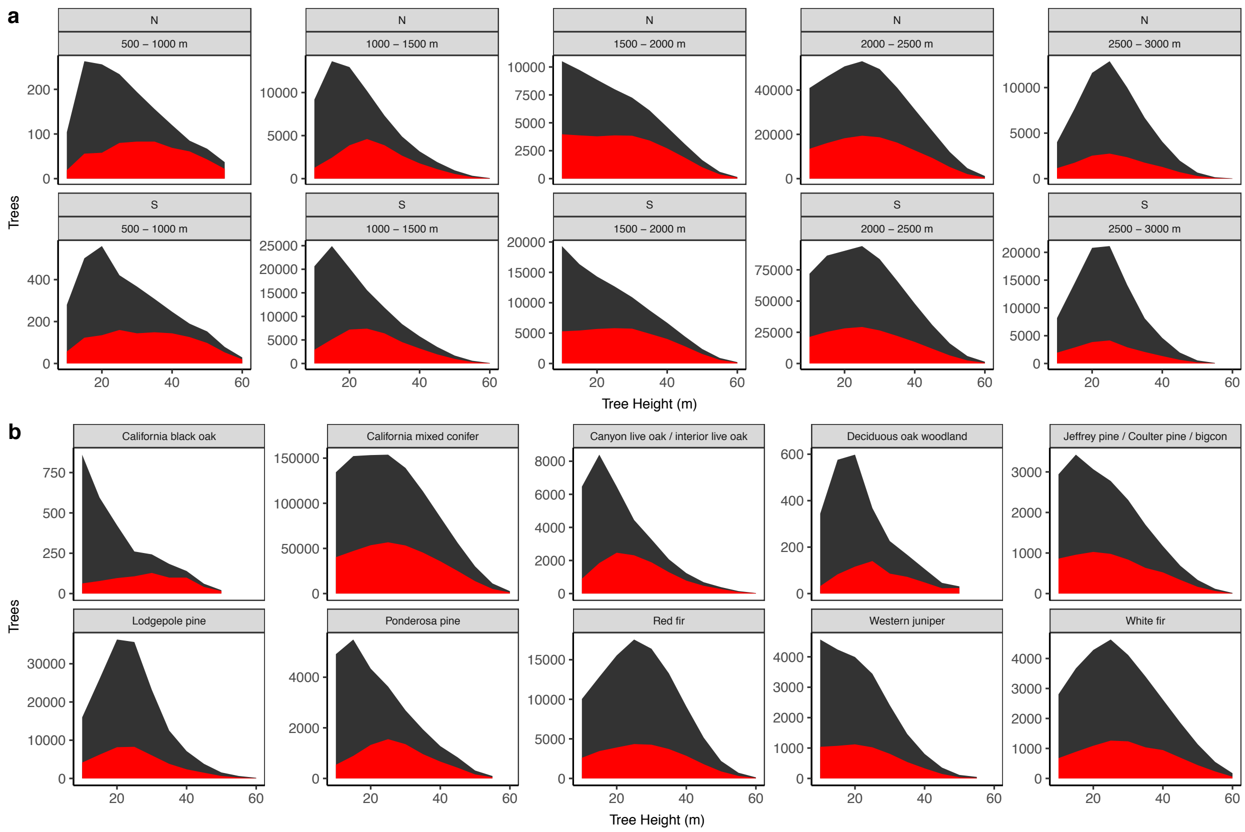

Supplementary Figure 1: Number of dead (red) and total (black) trees in the ~1.8 million tree dataset by subsets of [a] elevation and [b] forest type. Mortality percent, or the proportion of dead (red) to total (black) trees in an individual size class, controls for natural variation in tree abundance with respect to size, revealing the predominantly increasing mortality rate with increasing tree height. Note: y-axes are not fixed to improve visibility of the population distributions.

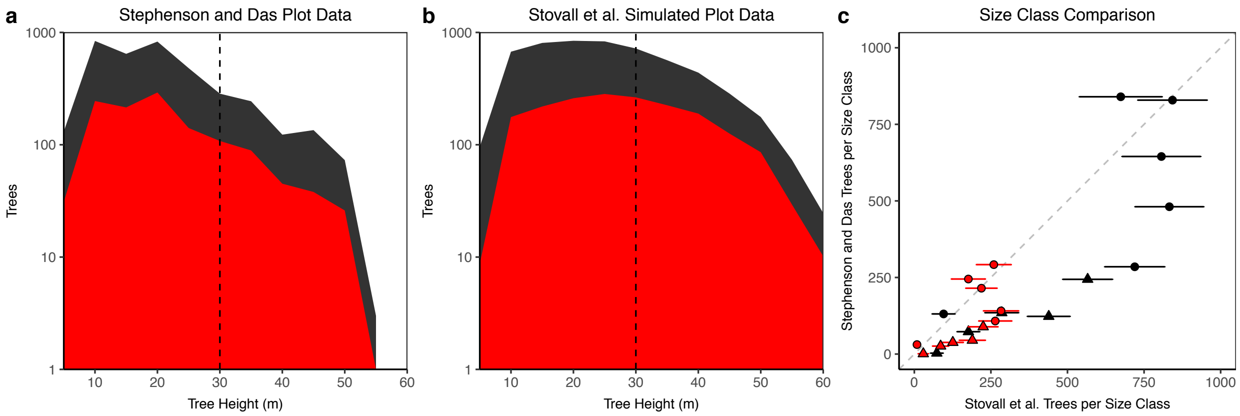

Supplementary Figure 2: Number of dead (red) and total (black) trees in the in the plot-based data from [a] Stephenson and Das and [b] the current study. On average, the simulated plots effectively recreate the field-based plot data captured from Stephenson and Das. Vertical black dashed lines [a-b] delineate the distribution into trees above and below 30 m in height. [c] Simulated plots identified more trees on average than the field-based methods, yet the distribution of total trees (black) and dead trees (red) with respect to small (circle) and large (triangle) size class is similar (grey dashed line is the 1:1: line and error bars are ± two standard deviations across 1000 simulations). Note: y-axes in [a] and [b] are log transformed to improve visibility of the population distributions.

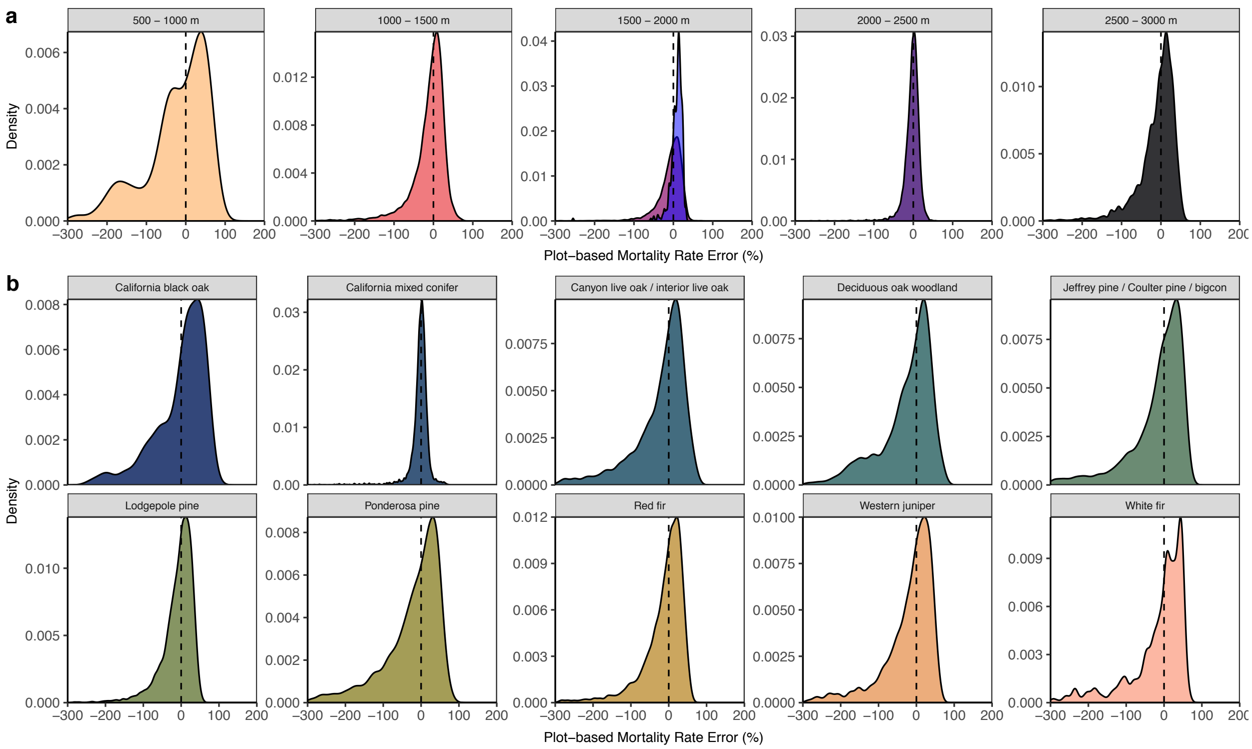

Supplementary Figure 3: Distribution of errors in the plot-based estimates of the landscape-level mortality rate across 1000 simulations of 89 0.1 ha plot locations stratified by [a] elevation and [b] forest type. A value of 0 (vertical dashed line) indicates the plot-based configuration correctly estimates the landscape-level mortality rate at a specific height class, while positive and negative errors indicate over and underestimates of mortality rate, respectively. The contiguous elevation band identical to Stephenson and Das is shown within the 1500 - 2000 m range in blue. In general, nearly every distribution has a long negative tail, where extreme underestimates of mortality rate occur, and a negatively biased mean (elevation = -10.3%; forest type = -25.1%). For example, across all forest types, errors in mortality rate greater than 50% occurred in 3.7% of the simulations, while underestimating more than 50% occurred in 22.4% of the simulations. The 5th percentile of error distribution was -388%, while the 95th percentile was 47%. In essence, plot-based methods are more likely to underestimate, as opposed to overestimate, mortality rate. Note: y-axes are not fixed to improve visibility of the error distributions.
