# Supplementary material for: Reply to “Height-related changes in forest composition explain increasing tree mortality with height during an extreme drought”
Source: Nat Commun. 2020 Jul 7;11:3401. doi: 10.1038/s41467-020-17214-4 (PMC7340790; doi:10.1038/s41467-020-17214-4)
Supplement: Supplementary file 2 — Reporting Summary [file 41467_2020_17214_MOESM2_ESM.pdf]

nature research

Corresponding author(s): Atticus E.L. Stovall

Last updated by author(s): May 20, 2020

Reporting Summary

Nature Research wishes to improve the reproducibility of the work that we publish. This form provides structure for consistency and transparency in reporting. For further information on Nature Research policies, see Authors & Referees and the Editorial Policy Checklist .

Statistics

For all statistical analyses, confirm that the following items are present in the figure legend, table legend, main text, or Methods section.

n/a	Confirmed	
☐	☒	The exact sample size (*n*) for each experimental group/condition, given as a discrete number and unit of measurement
☐	☒	A statement on whether measurements were taken from distinct samples or whether the same sample was measured repeatedly
☐	☒	The statistical test(s) used AND whether they are one- or two-sided<br>*Only common tests should be described solely by name; describe more complex techniques in the Methods section.*
☐	☒	A description of all covariates tested
☐	☒	A description of any assumptions or corrections, such as tests of normality and adjustment for multiple comparisons
☐	☒	A full description of the statistical parameters including central tendency (e.g. means) or other basic estimates (e.g. regression coefficient) AND variation (e.g. standard deviation) or associated estimates of uncertainty (e.g. confidence intervals)
☐	☒	For null hypothesis testing, the test statistic (e.g. *F*, *t*, *r*) with confidence intervals, effect sizes, degrees of freedom and *P* value noted<br>*Give P values as exact values whenever suitable.*
☒	☐	For Bayesian analysis, information on the choice of priors and Markov chain Monte Carlo settings
☒	☐	For hierarchical and complex designs, identification of the appropriate level for tests and full reporting of outcomes
☒	☐	Estimates of effect sizes (e.g. Cohen's *d*, Pearson's *r*), indicating how they were calculated

Our web collection on statistics for biologists contains articles on many of the points above.

Software and code

Policy information about availability of computer code

Data collection	All analysis is based on the original LiDAR-derived tree-level dataset (https://figshare.com/articles/CA_lidar_tree_mortality/7609193). The only additional data source used here is the USFS Forest Inventory and Analysis (FIA) forest type map (https://data.fs.usda.gov/geodata/rastergateway/forest_type/). Stratified random plot sampling schemes were developed using the grts function in the spsurvey R package (v4.1.1). We linked tree crowns to specific forest types with the extract function in the raster package (v3.0-12).
Data analysis	All data analysis was carried out in R using the base functions.

For manuscripts utilizing custom algorithms or software that are central to the research but not yet described in published literature, software must be made available to editors/reviewers. We strongly encourage code deposition in a community repository (e.g. GitHub). See the Nature Research guidelines for submitting code & software for further information.

Data

Policy information about availability of data

All manuscripts must include a data availability statement. This statement should provide the following information, where applicable:

- Accession codes, unique identifiers, or web links for publicly available datasets
- A list of figures that have associated raw data
- A description of any restrictions on data availability

The tree-level data generated from the LiDAR data along with associated environmental data are hosted through the following link: https://figshare.com/s/61098e084649e771ff03.

The USFS Forest Inventory and Analysis (FIA) forest type map is available at: https://data.fs.usda.gov/geodata/rastergateway/forest_type/

Field-specific reporting

Please select the one below that is the best fit for your research. If you are not sure, read the appropriate sections before making your selection.

☐ Life sciences ☐ Behavioural & social sciences ☒ Ecological, evolutionary & environmental sciences

For a reference copy of the document with all sections, see nature.com/documents/nr-reporting-summary-flat.pdf

Ecological, evolutionary & environmental sciences study design

All studies must disclose on these points even when the disclosure is negative.

Study description	Mapping location, size, and mortality of over 1.8 million trees in the Sierra Nevada mountains to determine the role of forest type and of topographic position on tree mortality during extreme drought.
Research sample	We relied on the tree-level data generated from NEON LiDAR in the original study. No additional processing of LiDAR was completed here. These derived tree-level data can be found at the following link: https://figshare.com/s/61098e084649e771ff03.
Sampling strategy	We simulated 1000 different plot placement strategies using our full tree-level dataset and an identical plot sampling approach as Stephenson and Das. We stratified 89 circular 0.1-ha plots with a probability-based design using the categories defined in our prior analysis: [i] topographic position and [ii] forest type. At each simulation step, 89 stratified plot locations are randomly selected using the grts function in the R package spsurvey (v4.1.1). Total sample locations are determined by the proportion of area attributed to each topographic or forest type class. A simulated plot is created as a 0.1-ha subset of tree crowns identified in our original study.
Data collection	We relied on the previously created tree-level data generated from the LiDAR data and hosted through the following link: https://figshare.com/s/61098e084649e771ff03.<br><br>The USFS Forest Inventory and Analysis (FIA) forest type map was downloaded at https://data.fs.usda.gov/geodata/rastergateway/forest_type/
Timing and spatial scale	The total study area spanned 40,854 ha and corresponded with two LiDAR acquisitions that took place in 06-09-2013 and 06-17-2013. Mortality estimates were derived from NAIP imagery collected on 07-23-14 and 07-25-16.
Data exclusions	We excluded forest types that did not cover a continuous range of height classes (less than 3) and did not cover a substantial portion of the study area.
Reproducibility	We have simplified the analysis conducted in R into a pipeline of processing scripts that produce the final tree-level dataset. We will make the analysis publicly available on GitHub and upon request.
Randomization	We selected 89 random stratified plot locations randomly with the grts function in the R package spsurvey (v4.1.1). We stratified by 500 m elevation bin and the USFS Forest Inventory and Analysis (FIA) forest type map (https://data.fs.usda.gov/geodata/rastergateway/forest_type/)
Blinding	Blinding was not relevant to this study because we relied on an automated sampling method.

Did the study involve field work? ☐ Yes ☒ No

Reporting for specific materials, systems and methods

We require information from authors about some types of materials, experimental systems and methods used in many studies. Here, indicate whether each material, system or method listed is relevant to your study. If you are not sure if a list item applies to your research, read the appropriate section before selecting a response.

Materials & experimental systems

n/a	Involved in the study	
☒	☐	Antibodies
☒	☐	Eukaryotic cell lines
☒	☐	Palaeontology
☒	☐	Animals and other organisms
☒	☐	Human research participants
☒	☐	Clinical data

Methods

n/a	Involved in the study	
☒	☐	ChIP-seq
☒	☐	Flow cytometry
☒	☐	MRI-based neuroimaging
